# Different impacts of the zero-markup drug policy on county general and traditional Chinese medicine hospitals: evidence from Shandong province, China

**DOI:** 10.1186/s12939-020-01326-w

**Published:** 2020-12-10

**Authors:** Xiaofeng Jiang, Ping He, Dawei Zhu, Xuefeng Shi, Qingyue Meng

**Affiliations:** 1 Center for Health Management and Policy Research, School of Public Health, Cheeloo College of Medicine, Shandong University, No. 44 Wen Hua Xi Lu, Lixia District, Jinan, 250012 China; 2 NHC Key Lab of Health Economics and Policy Research, Shandong University, No. 44 Wen Hua Xi Lu, Lixia District, Jinan, 250012 China; 3 Qilu Hospital of Shandong University, No. 107 Wen Hua Xi Lu, Lixia District, Jinan, 250012 China; 4 China Center for Health Development Studies, Peking University, 38 Xueyuan Road, Haidian District, Beijing, 100191 China; 5 School of Management, Beijing University of Chinese Medicine, No. 11, Bei San Huan Dong Lu, Chaoyang District, Beijing, 100029 China

**Keywords:** Zero-markup drug policy, County general hospitals, County traditional Chinese medicine hospitals, Dynamic effects, Difference-in-difference

## Abstract

**Background:**

As a key part of the new round of health reform, the zero-markup drug policy (ZMDP) removed the profit margins of drug sales at public health care facilities, and had some effects to the operation of these institutions. This study aims to assess whether the ZMDP has different impacts between county general and traditional Chinese medicine (TCM) hospitals.

**Methods:**

We obtained longitudinal data from all county general and TCM hospitals of Shandong province in 2007–2017. We used difference-in-difference (DID) method to identify the overall and dynamic effects of the ZMDP.

**Results:**

On average, after the implementation of the ZMDP, the share of revenue from medicine sales reduced by 16.47 and 10.42%, the revenue from medicine sales reduced by 24.04 and 11.58%, in county general and TCM hospitals, respectively. The gross revenue reduced by 5.07% in county general hospitals. The number of annual outpatient visits reduced by 11.22% in county TCM hospitals. Government subsidies increased by 199.22 and 89.3% in county general and TCM hospitals, respectively. The ZMDP reform was not significantly associated with the revenue and expenditure surplus, the number of annual outpatient visits and the number of annual inpatient visits in county general hospitals, the gross revenue, the revenue and expenditure surplus and the number of annual inpatient visits in county TCM hospitals. In terms of dynamic effects, the share of revenue from medicine sales, revenue from medicine sales, and gross revenue decreased by 20.20, 32.58 and 6.08% respectively, and up to 28.53, 63.89 and 17.94% after adoption, while government subsidies increased by around 170 to 200% in county general hospitals. The number of annual outpatient visits decreased by 9.70% and up to 18.84% in county TCM hospitals.

**Conclusion:**

The ZMDP achieved its some initial goals of removing the profits from western medicines in county hospitals’ revenue without disrupting the normal operation, and had different impacts between county general and TCM hospitals. Meanwhile, some unintended consequences were also recognized through the analysis, such as the decline of the utilization of the TCM.

## Introduction

High growth rates of healthcare expenditures have become a major concern worldwide [1, 2]. Therefore, lots of policies were implemented to control the unreasonable growth of healthcare expenditures. Many countries have implemented various policies to control the growth of healthcare expenditures; among these measures, a frequently used policy instrument is changing prices to reduce price-cost margins faced by healthcare providers [3]. Pricing policy is a frequently used instrument for this purpose by influencing behaviors of both health providers and users [4, 5]. In response to pricing policy, physicians might increase the volume and intensity of medical services with higher price-cost margins to compensate for revenue losses from the lower drug price regulations. Therefore, the impacts of fee changes on healthcare expenditure are not definitively known in theory, and empirical studies are needed [3]. The impacts of changing price-cost margins on health expenditure are examined, most of them from developed counties [4, 6]. Evidence from developing countries is limited even though those countries are facing similar challenges in containing rapid growth of healthcare expenditures and need studies for supporting policy options [2].

Reducing over-medicines through correcting distorted incentives is crucial for cost containment. The Chinese government launched a new round of health reform in 2009, aiming to achieve the universal health coverage by 2020. The zero-markup drug policy(ZMDP), as the key part of the reform [7], is considered as a key measure to address over prescriptions of drugs through removing drug markups for revenue generation for healthcare providers [8]. Similar with other countries, most of pharmaceuticals are sold in China at regulated prices since 1997 [9, 10]. For hospitals, retail prices of western medicines should be theoretically equal the provider’s procurement price plus a 15% profit margin [10]. The ZMDP refers to the removal of 15% profit margins of drug sales at public health care facilities that had been used as one of sources for financing those health providers over a half century [7, 10]. The purpose of the ZMDP is to reduce patients’ financial burden and change the profit-seeking behaviors of public health care institutions [8]. In the county level, health care is delivered via a three-tiered system [11], comprising village clinics, township health centers and county hospitals. Generally, there are one general hospital and one traditional Chinese medicine (TCM) hospital in each county [12]. The county general hospitals mainly provide western medicine and a minority of herbal medicines, otherwise county TCM hospitals mainly provide TCM medicines. The ZMDP was first implemented at primary care institutions in 2010, then expanded to county public hospitals in 2012, and then expanded to all county public hospitals in 2015 in Shandong Province, one of the most populated provinces in China. In county hospitals, the ZMDP was implemented through the electronic information system at the certain time by the health administrative departments and price departments. The ZMDP was not applied to herbal medicines to support the utilization of the TCM [8, 13], which implied that the policy may exert different impacts between county general and TCM hospitals.

County TCM hospitals provide both herbal medicines and western medicines [14]. With the ZMDP, county TCM hospitals could try to increase the volumes and intensity of TCM services with higher price-cost margins to compensate for revenue losses. The revenue from Chinese medicines as a share of total drug revenue in county TCM hospitals and county general hospitals were 36 and 6%, respectively [13], which implied that county TCM hospitals had higher capacities than county general hospitals to shift the revenue generation from western medicines to Chinese medicines [15]. The different structure of revenue generation between county general hospitals and TCM hospitals provides an opportunity for analyzing the effects of the ZMDP. Some studies have examined the effects of the ZMDP. One study compared the different impacts of the ZMDP on western medicines and Chinese medicines in county TCM hospitals [8], but it still did not compare the difference between county general and TCM hospitals. Furthermore, for policy-makers and similar settings where the traditional medicine plays an important role in their health system, it is important to know the difference in the impacts of the ZMDP between county general and TCM hospitals. Understanding the difference in the impacts of the ZMDP between county general and TCM hospitals will be useful for policy-makers to make more effective policies for addressing the cost shifting behaviors of health providers.

To fill the gap in this field on the evaluation of the potential different effects, we conducted a study to compare the impacts of the ZMDP between county general and TCM hospitals in Shandong province during 2007–2017.

## Methods

### Data sources

This study obtained data from the Financial Annual Report Data of County-level Public Hospitals which was collected by the information center of Health and Family Planning Commission of Shandong Province (now renamed Health Commission of Shandong Province). The data contained information on human resources, hospital scales, annual revenues, outpatient and inpatient services, etc.

**Fig. 1:**
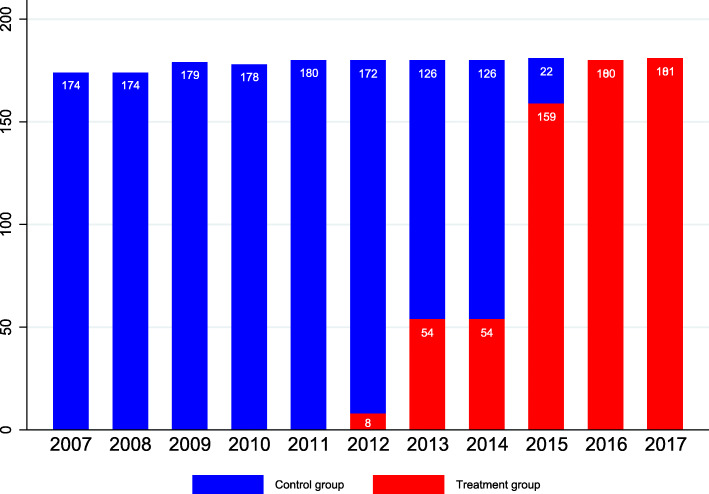
Number of county hospitals and the inception of the ZMDP

The information on the timing of the ZMDP implementation was mainly from the website of city health bureau, the government report or official documents. In total, we obtained the launch year and month of 97 counties in Shandong province. As shown in Fig. 1, there were 174 county general and TCM hospitals in Shandong province in 2007. In 2012, eight hospitals began to implement the ZMDP, accounting for less than 5% of the total number of county public hospitals. In 2016, the policy covered all 181 hospitals. In total, we included 1967 pieces of hospital-year information during 2007–2017 to establish unbalanced panel data for analysis.

### Outcome measures

According to the goal of the ZMDP, the outcome variables in this study were revenue from medicine sales, the share of revenue from medicine sales (divide the amount of revenue from medicine by total revenue except government subsidies), revenue from medical care services, government subsidies, which reflected the structure of the revenue of county hospitals [8, 13, 16]. The outcome variables also included revenue and expenditure surplus, gross revenue, the number of annual outpatient and inpatient visits, which reflected the impacts of the policy on the overall operation of county hospitals.

### Treatment definitions

To define treatment groups, we merged the county hospital data with the county-by-year indicator of whether the ZMDP had been adopted. The treatment definition in the present study was a binary classification of treatment shown in Fig. 1: “treated” if the county hospital implemented the ZMDP; otherwise, “untreated”. To separate the short-run from long-run effects, we tested whether the effects of the ZMDP were immediate or took time to build, and whether the effects were transient or lasting. From this perspective, we used “duration” to identify whether the treatment effects changed over time.

### Summary statistics

Difference-in-difference (DID) models were used to assess the different impacts of the ZMDP between county general and TCM hospitals. The DID, or more generally, fixed-effects model, is:

1 $$ \log \left({Y}_{it}\right)=\alpha +{\lambda}_1\times {Policy}_{it}+{\lambda}_2\times {Policy}_{it}\times {TCM}_i+\beta {X}_{it}+{\mu}_i+{\gamma}_t+{\delta}_{mt}+{\varepsilon}_{it}. $$

Where *Y*_*it*_ represented a series of the outcome variables in hospital (*i*) and year (*t*). Logarithmic transformations were performed on all outcome variables to adjust for the right-skewed data. The key explanatory variable was *Policy*_*it*_, which indicated whether hospital *i* had implemented the ZMDP by year *t*. The other explanatory variable of interest, *TCM*, denoted whether hospital *i* was a TCM hospital. The coefficient *λ*_1_ was the average treatment effect on the outcome at county general hospitals, and *λ*_2_ was the differential impact of the policy implementation on county TCM hospitals, *Y*_*it*_. Control variables, namely *X*_*it*_, included the number of medical staffs, hospital beds and were also estimated in logs. All estimates included a vector of hospital’s individual fixed effects(*μ*_*i*_)that control for mean differences across hospitals, and year dummies(*γ*_*t*_)that controlled for flexible year effects common to all hospitals, and county-specific time trend (*δ*_*mt*_) that relaxed the common-trend assumption by allowing different counties to follow different trends. *ε*_*it*_ referred to the error term. Standard errors were clustered at the county level.

In addition, to understand how quickly *Y*_*it*_ grew or decreased after implementing the policy and whether this impact accelerated, stabilized or reverted, we estimated the dynamic effects of the ZMDP by using 3 “lags” of the treatment effect:

2 $$ {\displaystyle \begin{array}{l}\log \left({Y}_{it}\right)=\alpha +{\sum}_{j=0}^3{\lambda}_{j1}{Duration}_{it\left(t=k+j\right)}+{\sum}_{j=0}^3{\lambda}_{j2}{Duration}_{it\left(t=k+j\right)}\times {TCM}_i+\\ {}\beta {X}_{it}+{\mu}_i+{\gamma}_t+{\delta}_{mt}+{\varepsilon}_{it}\end{array}}. $$

where we added four “treatment variables”, *Duration*_*it*(*t* = *k* + *j*)_, for years 0–3 after adopting the ZMDP. *k* was the time at which the reform was being switched on hospital *i*. Of these four indicators, we noted that the first three took on the value 1 only in the relevant year, while the final variable was equal to 1 in each year, starting with the third year of implementation.

The main challenge for DID estimation was the “parallel trends” assumption which required that in the absence of treatment, the difference between the ‘treatment’ and ‘control’ group was constant over time. Following previous researches [8, 17], we tested the “parallel trends” assumption by assuming that the reforms happened 2 or 3 years ahead. If the DID estimate was insignificant or had conflicting signs, which meant the trends were parallel, then our DID estimates were unlikely to be biased.

A *p*-value of less than 0.05 was considered statistically significant. The software Stata version 15 for Windows (Stata Corp, College Station, TX, USA) was used for the statistical analysis.

## Results

### Characteristics of the study hospitals

Table 1 summarized the descriptive results for county-level hospitals. Among 1967 hospital-year observations, the average share of revenue from medicine sales was 44.20%. The average government subsidies per hospital was 6.87 million CNY (0.97 million USD). The average gross revenue per hospital was 155.38 million CNY (21.84 million USD), and the average revenue and expenditure surplus was 5.60 million CNY (0.79 million USD). The average number of annual outpatient and inpatient visits per hospital was 243.83 and 20.21 thousand, respectively. On average, a county public hospital had 539 medical professionals and 511 hospital beds.

**Table 1:** Characteristics of the study hospitals (*n* = 19,089)

Variable	Mean	SD
**Outcome variables**		
Share of revenue from medicine sales (%)	44.20	9.63
Revenue from medicine sales (Million Yuan)	60.66	61.34
Revenue from medical care service (Million Yuan)	85.66	97.02
Government subsidies (Million Yuan)	6.87	12.07
Revenue and expenditure surplus (Million Yuan)	5.60	18.79
Gross revenue (Million Yuan)	155.38	162.72
Number of annual outpatient visits (Thousand)	243.83	221.39
Number of annual inpatient visits (Thousand)	20.21	16.67
**Control variables**		
Number of medical staff	539.24	383.50
Number of hospital beds	510.79	376.50

### Overall effect of the ZMDP at county-level hospitals

**Fig. 2:**
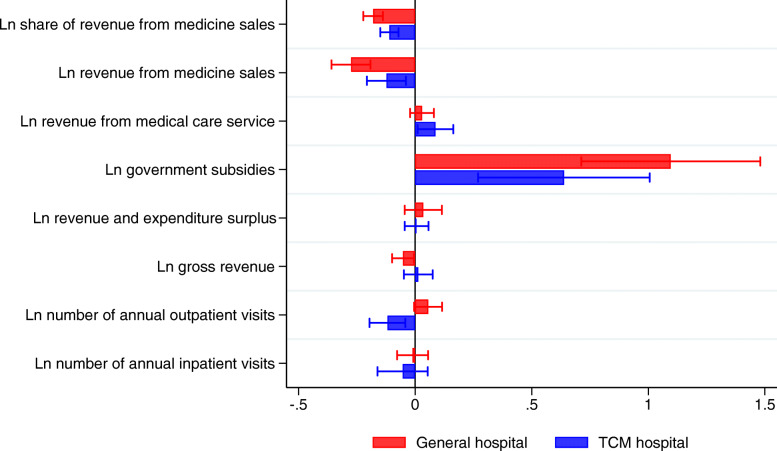
Overall effect of the ZMDP at county hospitals by hospital types

Figure 2 displayed the point estimates and 95% confidence intervals on the average effect of the ZMDP at county general and TCM hospitals. Corresponding regression results were presented in Table 2.

**Table 2:** Estimated average impact of the ZMDP on county hospitals by hospital types

Variables	Ln share of revenue from medicine sales	Ln revenue from medicine sales	Ln revenue from medical care service	Ln government subsidies	Ln revenue and expenditure surplus	Ln gross revenue	Ln number of annual outpatient visits	Ln number of annual inpatient visits
Policy	− 0.180*** (0.021)	− 0.275*** (0.043)	0.029 (0.026)	1.096*** (0.196)	0.035 (0.041)	−0.052* (0.024)	0.056 (0.031)	−0.011 (0.034)
Policy *TCM	0.070** (0.021)	0.152** (0.049)	0.058 (0.037)	−0.458** (0.159)	−0.029 (0.017)	0.066 (0.038)	−0.175*** (0.044)	− 0.043 (0.056)
Observations	1967	1967	1967	1912	1966	1967	1967	1965
R-squared	0.563	0.781	0.912	0.355	0.006	0.893	0.596	0.594

*p < 0.05, ** *p* < 0.01, *** *p* < 0.001

For the revenue structure, the ZMDP reform reduced the share of revenue from medicine sales in both county general (16.47% = 1-exp^− 0.180^, *P* < 0.001) and TCM hospitals (10.42% = 1-exp^− 0.110^, *P* < 0.001). After the implementation of the ZMDP, the revenue from medicine sales decreased by 24.04% (1-exp ^− 0.275^, *P* < 0.001) and 11.58% (1-exp^-0.1231^, *P* < 0.01) in county general and TCM hospitals, respectively. The government subsidies increased by 199.22% (exp^1.096^–1, *P* < 0.001) and 89.3% (exp^0.638^–1, *P* < 0.001) in county general and TCM hospitals, respectively. The ZMDP reform, on average, significantly reduced gross revenue in county general hospitals (5.07% = 1-exp^-0.052^, *P* < 0.05) while there was an insignificant increase of gross revenue in county TCM hospitals. The ZMDP did not have a significant impact on the revenue and expenditure surplus, the number of annual outpatient visits and the number of annual inpatient visits in county general hospitals. However, the number of annual outpatient visits in county TCM hospitals had decreased (11.22% = 1-exp^-0.119^, *P* < 0.01) after the implementation of the ZMDP.

In total, the ZMDP had significantly different impacts between county general and TCM hospitals in the share of revenue from medicine sales, the revenue from medicine sales, the government subsidies, the gross revenue and the annual number of outpatient visits. It’s worth noting that the identified assumption of these DID estimator was that time trends of these outcomes would have been the same in both implementing and non-implementing hospitals pre-reform.

### Dynamic effects of the ZMDP

**Fig. 3:**
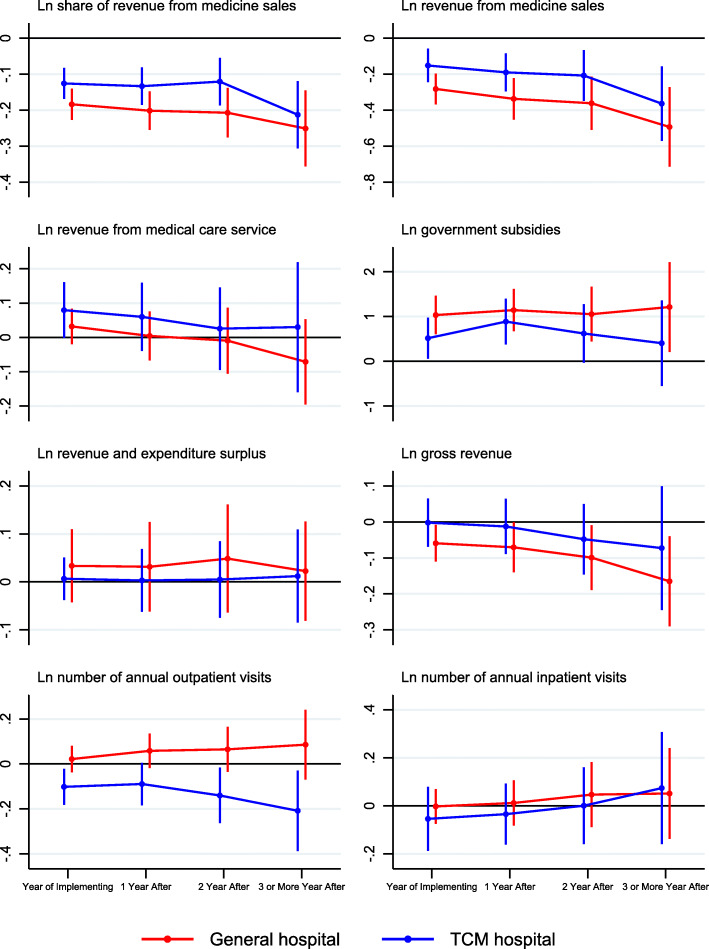
Dynamic effect of the ZMDP at county hospitals by hospital types

Figure 3, which was based on Table 3, presented the estimated dynamic impacts of the ZMDP reform on outcomes. More precisely, the red solid line traced coefficient estimates of *λ*_*j*1_ from estimation of Eq. (2), which represented the long-term effects of the ZMDP reform on county general hospitals, and the blue solid line traced the sum of *λ*_*j*1_ and *λ*_*j*2_, which represented the long-term effects of the ZMDP reform on county TCM hospitals.

**Table 3:** Dynamic effect of the ZMDP at county hospitals by hospital types

Variables	Ln share of revenue from medicine sales	Ln revenue from medicine sales	Ln revenue from medical care service	Ln government subsidies	Ln revenue and expenditure surplus	Ln gross revenue	Ln number of annual outpatient visits	Ln number of annual inpatient visits
the adoption year	−0.184*** (0.022)	−0.282*** (0.044)	0.032 (0.027)	1.032*** (0.219)	0.034 (0.039)	−0.059* (0.026)	0.022 (0.030)	−0.003 (0.037)
1 year after adoption	−0.202*** (0.027)	− 0.337*** (0.059)	0.004 (0.036)	1.141*** (0.240)	0.032 (0.048)	−0.071* (0.035)	0.058 (0.039)	0.012 (0.048)
2 years after adoption	−0.207*** (0.035)	−0.362*** (0.076)	− 0.010 (0.049)	1.051** (0.313)	0.049 (0.057)	−0.099* (0.046)	0.065 (0.051)	0.047 (0.069)
3 or more years after adoption	−0.251*** (0.054)	−0.494*** (0.113)	− 0.071 (0.063)	1.211* (0.512)	0.022 (0.053)	−0.165* (0.064)	0.086 (0.079)	0.051 (0.096)
the adoption year *TCM	0.058* (0.024)	0.130* (0.055)	0.048 (0.039)	−0.517** (0.177)	−0.027 (0.019)	0.057 (0.043)	−0.124** (0.043)	− 0.052 (0.077)
1 year after adoption *TCM	0.068** (0.023)	0.147** (0.051)	0.056 (0.041)	−0.257 (0.180)	−0.028 (0.017)	0.058 (0.041)	−0.148** (0.046)	− 0.047 (0.050)
2 years after adoption *TCM	0.087*** (0.025)	0.154** (0.047)	0.035 (0.040)	−0.432* (0.205)	−0.044* (0.020)	0.051 (0.040)	−0.205*** (0.054)	− 0.046 (0.056)
3 or more years after adoption *TCM	0.038 (0.032)	0.130 (0.082)	0.101 (0.067)	−0.808 (0.445)	−0.010 (0.014)	0.092 (0.066)	−0.294*** (0.077)	0.023 (0.067)
Observations	1967	1967	1967	1912	1966	1967	1967	1965
R-squared	0.568	0.784	0.913	0.356	0.006	0.894	0.599	0.596

*** *p* < 0.001, ** *p* < 0.01, * *p* < 0.05

Once the ZMDP reform took effective at county general hospitals, the share of revenue form medicine sales, revenue from medicine sales, and gross revenue decreased by 20.20% (1-exp^-0.184^, *P* < 0.001), 32.58% (1-exp^-0.282^, *P* < 0.001), and 6.08% (1-exp^-0.059^, *P* < 0.05), respectively, and up to 28.53% (1-exp^-0.251^, *P* < 0.001), 63.89% (1-exp^-0.494^, *P* < 0.001), and 17.94% (1-exp^− 0.165^, *P* < 0.05) in 3 or more years after adoption. The ZMDP reform substantially increased government subsidies, increasing by 180.67% (exp^1.032^–1, *P* < 0.001) in the adoption year, and fluctuating around 170 to 200% in the subsequent years.

When it came to county TCM hospitals, in the adoption year, the ZMDP reform reduced the number of annual outpatient visits by 9.70% (1-exp^-0.102^, *P* < 0.05), and up to 18.84% (1-exp^-0.209^, *P* < 0.05) in 3 or more years after adoption. However, the ZMDP did not have a significant effect on gross revenue. The impacts on other outcomes were similar to that of county general hospitals. Again, the identified assumption of these DID estimator was that time trends of these outcomes would have been the same in both implementing and non-implementing hospitals pre-reform.

### Robustness check

A potential challenge to the DID strategy was that differential changes between implementing and non-implementing hospitals may be driven by preexisting differences in the time trends of the outcomes. To address this issue, we constructed two pseudo-reform models by assuming that the reform happened 2 (pseudo-reform A model) or 3 (pseudo-reform B model) years ahead. Table 4 showed the test results of the parallel trend assumption on all outcomes. We found that the relevant estimates of placebo tests were either insignificant or have conflicting signs, which suggested that our primary findings were driven by the ZMDP rather than by some unobservable factors.

**Table 4:** Parallel trend test assuming the reform happened 2 or 3 years ahead

	Ln share of revenue from medicine sales	Ln revenue from medicine sales	Ln revenue from medical care service	Ln government subsidies	Ln revenue and expenditure surplus	Ln gross revenue	Ln number of annual outpatient visits	Ln number of annual inpatient visits
Pseudo-reform A (Assumed 2 years ahead)	0.014 (0.016)	0.001 (0.031)	−0.019 (0.016)	−0.180 (0.159)	−0.081 (0.082)	− 0.035 (0.021)	0.012 (0.031)	− 0.017 (0.035)
Observations	1967	1967	1967	1912	1966	1967	1967	1965
R-squared	0.518	0.771	0.912	0.338	0.009	0.893	0.584	0.594
Pseudo-reform B (Assumed 3 years ahead)	0.032 (0.012)*	0.049 (0.025)*	−0.000 (0.024)	−0.360 (0.169)*	0.015 (0.010)	−0.009 (0.021)	0.020 (0.029)	0.005 (0.034)
Observations	1967	1967	1967	1912	1966	1967	1967	1965
R-squared	0.520	0.771	0.912	0.340	0.005	0.893	0.584	0.593

All models adjusted for individual fixed effects, yearly fixed effects, medical staffs and hospital beds. Standard errors in parentheses

*** *p* < 0.001, ** *p* < 0.01, * *p* < 0.05

## Discussion

Using hospital-based longitudinal data from all county public hospitals in Shandong province, we conducted a study to compare whether there was a difference in the impacts of the ZMDP between county general and TCM hospitals. We found that the revenue and expenditure surplus and the number of inpatients visits did not change, the revenue from medicine sales and the share of revenue from medicine sales fell off, government subsidies increased in county general and TCM hospitals after the launch of the ZMDP. Meanwhile, we also observed that the gross revenue decreased significantly in county general hospitals but did not change in county TCM hospitals, the number of outpatient visits did not change in county general hospitals but decreased in county TCM hospitals after the introduction of the ZMDP.

As expected, after the ZMDP was implemented, both the revenue from medicine sales and the share of revenue from medicine sales decreased dramatically in both county general and TCM hospitals, mainly due to the fact that medicine sales accounted for a big proportion of revenue in both institutions. With the ZMDP, county general and TCM hospitals may have no incentive to prescribe drugs for the revenue purpose, implying that the ZMDP was most likely effective in controlling drug expenditures. If the prescribing practice kept the same before and after the introduction of the ZMDP, the revenue from medicine sales would reduce by 15% or less, since herbal medicines are exempted from the ZMDP. In this study, the revenue from medicine sales decreased by 24.04% in county general hospitals which is more than 15%. This indicated that ZMDP may reduce the incentives to liberally prescribe drugs. Similar findings were found in a study at county general hospitals in Shanxi province [18], a study at county TCM hospitals throughout the country [8], and a study at township health centers in three provinces of China [19].

The dynamic estimates showed that the revenue from medicine sales and the share of revenue from medicine sales had continued to decrease and maintained a downward momentum since the implementation of the ZDMP. The extent of decline on the revenue from medicine sales and the share of revenue from medicine sales in county general hospitals were greater than that in county TCM hospitals and the tendency kept on in 3 years after the ZMDP was launched. This was likely because the share of western medicines was greater in county general hospitals than that in county TCM hospitals [20]. Thus, the revenue from medicine sales and the share of revenue from medicine sales experienced larger decrease in county general hospitals than that in county TCM hospitals.

This study showed the ZMDP did not have a significant impact on the revenue from medical care services. In theory, if county general and TCM hospitals provide more medical care services to compensate the income loss from drugs, the revenue from medical care services would increase with the ZMDP. There were several reasons why the revenue from medical services did not change after the ZMDP. First, the government had taken three main measures, including increasing prices of professional services of health care, increasing government subsidies, and strengthening hospital financial management, to compensate the income loss due to the ZMDP [21]. Many local governments did not effectively increase the prices of medical services in time. Consequently, the revenue from the medical services did not change much before and after the implementation of the ZMDP. Second, when raising the prices of medical services, which could reflect the labor value of physicians, lab test, imaging screening and medical consumables would decrease in the same time [8]. In this study, the revenue from medical care services contained the revenue from lab test, imaging screening and medical consumables, and we could not separate them. Therefore, the revenue from the medical services may not change much before and after the implementation of the ZMDP. To identify the comprehensive effects of the ZMDP, future studies should use more detailed data to investigate which part of medical services was changed, for example, whether the lab test and imaging screening expending increased.

We found government subsidies substantially increased after the launch of the ZMDP in county hospitals. This was highly in accordance with previous studies [8, 16, 19]. In county general hospitals, government subsidies increased and kept an upward momentum after the ZMDP was introduced. In county TCM hospitals, government subsidies increased in the first 2 years after the implementation of the ZMDP. In the third and fourth year, government subsidies did not change much. The increase of government subsidies in county general hospitals was higher than that in county TCM hospitals and the gap had been enlarged over time. This was likely because of the following reasons. First, the share of revenue from western medicines sales in gross revenue was greater in county general hospitals than that in county TCM hospitals. The loss of gross revenue in county general hospitals due to the ZMDP was more than that in county TCM hospitals. Second, the revenue from herbal medicines which were not adapted to the ZDMP accounted for a big proportion in gross revenue in county TCM hospitals. Third, the prices of the TCM diagnosis and treatment technology might increase after the implementation of the ZMDP. Therefore, the loss of gross revenue in county TCM hospitals was relatively much less than that in county general hospitals, which led to less government subsidies.

We found the number of outpatient visits in county TCM hospitals decreased after the ZMDP. This was likely due to two reasons. First, relative to western medicines, the increase in relative prices of Chinese herbal drugs would likely reduce the patients’ demand for the TCM [8]. Second, there was a view that the TCM had started to move from mainstream to marginal hospital care in China [22]. To strengthen the utilization and development of the TCM, the ZMDP should be implemented in all traditional medicines including herbals to attract more people to use TCM services.

We found gross revenue in county general hospitals decreased, but did not change much in county TCM hospitals after the ZMDP. There were several potential reasons for the decrease of gross revenue in county general hospitals. First, the share of revenue from western medicines sales in gross revenue accounted for a greater proportion in county general hospitals than that in county TCM hospitals. Second, as we mentioned before, the revenue from medical care services in county general hospitals did not change much before and after the ZMDP. Third, the increase in government subsidies may not be enough to offset the decline in the revenue from western medicines sales in county general hospitals. This implied that the policymakers should consider the difference between county general and TCM hospitals and make corresponding complementary policies for them. For example, to keep the consistent policy implementation between county general and TCM hospitals, whether the Chinese herbal medicines should also be adapted to the ZMDP is warranted to be considered. In addition, it is important for the local government, to further implement the policy, to increase the prices of the medical care services to fully offset the revenue loss for the county hospitals.

In the present study, we took the revenue and expenditure surplus as an important variable, which was firstly studied and would be the supplement to the existing studies. If the revenue and expenditure surplus experienced a sharp decrease after the ZMDP, whether the ZMDP could take effect would be a big question. This study demonstrated the ZMDP had no significant impact on revenue and expenditure surplus in both county general and TCM hospitals, suggesting the ZMDP had no adverse impacts on the normal operation of county hospitals, which was vital to the reform. In the next step for both county general and TCM hospitals, it is important to further strengthen hospital management, improve operation efficiency and reduce operation costs.

Several policy implications should be considered. First, the impacts of the ZMDP had some differences between county general and TCM hospitals. Therefore, the policymakers should enact different policies for them respectively. Second, despite the ZMDP having significant impacts on the income structure of county hospitals, some effects showed a weakening trend over time, such as government subsidies to county TCM hospitals. Therefore, government subsidies should be strengthened to county TCM hospitals in the future, to assure the sustainability of the ZMDP. Third, for policy-makers in China and other countries, our findings suggest that changing prices for drugs and medical services may yield complicated outcomes, and they should consider both the demand and supply side. On the demand side, patients may increase the demand for drugs and services because of the decreased prices. On the supply side, physicians may increase the provision of other services with higher price-cost margins.

There were several limitations. First, while our tests of the DID parallel trends assumption and robustness checks showed good validity and consistency, other policies except the ZMDP during the same period might be associated with the changes of outcomes we found. Second, we have identified that the ZMDP had some effects on county hospitals, but we did not know whether the policy affected the behaviors of physicians and patients. Finally, due to data limitations, findings concluded from this study might not be totally generalized to the whole country.

## Conclusion

This is the first comparative study regarding the impacts of the ZMDP on county general and TCM hospitals. We found that the ZMDP achieved its initial goals of removing the revenue and expenditure surplus from western medicines in county hospitals’ revenue without disrupting their normal operation. Some unintended consequences should also be recognized. For example, the ZMDP reform decreased the utilization of the TCM and did not achieve the goal of protecting the TCM. Further studies should be conducted to explore whether the ZMDP impacted the behaviors of physicians and patients.

## Data Availability

The data that support the findings of this study are available from Health Commission of Shandong Province but restrictions apply to the availability of these data, which are used under license for the current study, and so are not publicly available. Data are however available from the authors upon reasonable request and with permission of Health Commission of Shandong Province.

## Abbreviations

ZMDP: Zero-markup drug policy;
TCM: Traditional Chinese medicine;
DID: Difference-in-difference
